# Transcranial direct current stimulation modulates efficiency of reading processes

**DOI:** 10.3389/fnhum.2015.00114

**Published:** 2015-03-16

**Authors:** Jennifer M. Thomson, Deniz Doruk, Bryan Mascio, Felipe Fregni, Carlo Cerruti

**Affiliations:** ^1^Harvard Graduate School of EducationCambridge, MA, USA; ^2^Spaulding Neuromodulation Center, Spaulding Rehabilitation Hospital, Harvard Medical SchoolBoston, MA, USA

**Keywords:** tDCS, phonological processing, reading efficiency, neuromodulation, temporoparietal junction

## Abstract

Transcranial direct current stimulation (tDCS) is a neuromodulatory technique that offers promise as an investigative method for understanding complex cognitive operations such as reading. This study explores the ability of a single session of tDCS to modulate reading efficiency and phonological processing performance within a group of healthy adults. Half the group received anodal or cathodal stimulation, on two separate days, of the left temporo-parietal junction while the other half received anodal or cathodal stimulation of the right homologue area. Pre- and post-stimulation assessment of reading efficiency and phonological processing was carried out. A larger pre-post difference in reading efficiency was found for participants who received right anodal stimulation compared to participants who received left anodal stimulation. Further, there was a significant post-stimulation increase in phonological processing speed following right hemisphere anodal stimulation. Implications for models of reading and reading impairment are discussed.

## Introduction

Being an efficient reader in today’s society greatly increases an individual’s chances of academic success and subsequent employment potential (Organisation for Economic Co-operation and Development (OECD), 2000; Achieve, 2005; ACT, 2005). Yet unlike the acquisition of oral language, learning to read requires years of explicit teaching and effortful practice in order to achieve mastery, with much individual variation in the final level of skill attained. Given the resources put into literacy education, there is a constant desire to optimize the process, and to reduce the gap between good and poor readers.

Neuroscience has been integral to this drive for knowledge. A key task for neuroimaging has been to identify areas of the brain that are important for the reading process. The act of reading comprises multiple subcomponents and the first task of reading in an alphabetic language is learning to link sounds and letters—i.e., decoding. Individuals subsequently gain increasing fluency in decoding, while steadily placing increasing resources into the act of comprehension (Snow et al., 1998). So far, the act of decoding and its pre-cursor skills have been the most accessible to neuroscientific study. Understanding decoding has also garnered particular attention in the wider research and educational community because unexpected difficulties with this task characterize developmental dyslexia, a specific difficulty with reading of neurobiological origin, which has a phonological deficit at its core (Gabrieli, 2009; Peterson and Pennington, 2012). Phonological processing is the ability to reflect upon and manipulate the component sounds of a word; including syllables, as well as phonemes, smaller sound units which often correspond to letters.

Functional imaging studies have converged in the last two decades in identifying a left-hemisphere lateralized reading network that implicates the inferior frontal as well as extensive posterior regions (Turkeltaub et al., 2003; Martin et al., 2015). Underactivation of these regions has also been reported in individuals who have persistent reading difficulties such as dyslexia (Rumsey et al., 1992; Horwitz et al., 1998; Paulesu et al., 2001; McCandliss and Noble, 2003; Hoeft et al., 2006; Shaywitz et al., 2006). In addition, research has reported overactivity of the left inferior frontal gyrus in dyslexia (McCandliss and Noble, 2003; Sandak et al., 2004), and less commonly, increased activity and connectivity in the right homologous regions, compared to typically-reading controls (Milne et al., 2002; Finn et al., 2014). It has been found that successful behavioral intervention targeting the phonological deficit in dyslexia is frequently accompanied by increases in “normalization” of functional activation of cortical and subcortical, predominantly left hemisphere areas thought to be implicated in the early stages of reading (see Barquero et al., 2014, for a review).

This body of knowledge raises intriguing questions. For example, if improved behavioral outcomes consistently correlates with an increase in related cortical activity, would using neuromodulation techniques to increase cortical activity result in parallel behavioral gains? A potential neuromodulation tool to help answer this question is transcranial direct current stimulation (tDCS). tDCS is a safe, noninvasive method of neuromodulation during which a weak direct current is applied via anodal (increasing) and cathodal (decreasing) electrodes strategically placed on the scalp. Modeling studies support the idea that current passes through the skull and changes the excitability of targeted brain areas (Bikson et al., 2012). Resting membrane potentials of local populations of neurons are modulated, impacting postsynaptic subthreshold membrane de/polarization and thus increasing or decreasing the likelihood that a stimulus of constant strength will cause the neurons to reach their activation threshold (Nitsche et al., 2003, 2008). The amount of current reaching cortical areas directly under tDCS electrodes is significant (Wagner et al., 2006, 2007) and tDCS has been successfully used to elicit functional changes in motor function, mood and language/cognitive function (Nitsche and Paulus, 2000; Brunoni et al., 2012). One 10-min session of anodal tDCS results in excitability shifts lasting greater than one hour (Nitsche and Paulus, 2001), with multiple sessions resulting in longer-lasting shifts (Fregni and Pascual-Leone, 2007).

tDCS has recently been applied to the domain of reading. In 2012 Turkeltaub et al. applied anodal stimulation to the left posterior temporal cortex (pTC) with concurrent cathodal stimulation at homologous right pTC in a group of typically-reading adults (Turkeltaub et al., 2012). This was compared to a sham condition in the same individuals, administered on a separate day and counterbalanced for order. After a single 20 min application of tDCS, higher reading scores were reported in the active condition, as compared to sham, as measured by the Test of Word Reading Efficiency (TOWRE; Torgesen et al., 1999) a standardized, timed, single word reading measure. This pattern did not hold for the whole group, but just in the lower performing subgroup.

Turkeltaub’s study is an important step forward, but also raises many additional questions. Firstly, because the reading measures were only administered post-stimulation, we are making an inference that the active vs. sham performance differences are directly due to the presence or absence of stimulation. Without pre-stimulation reading measures reported, the strength of this inference is diminished. Secondly, by using the right homologous pTC site for the positioning of the reference electrode (which acts to make stimulation to that area cathodal), it becomes difficult to know whether the locus of the effect is due to the anodal stimulation of the left hemisphere or the dual action of anodal stimulation to the left hemisphere and cathodal stimulation of the right hemisphere. Finally, given the complexity of the reading process, it is of interest to see the effect of stimulation on specific subcomponents of reading, to add further nuance to our understanding of the effects of tDCS on this skill. For these reasons, the current study was carried out.

We report here the findings for a group of healthy, typically-reading adults. Stimulation of left hemisphere and right hemisphere temporo-parietal junction was administered on separate days, however in each case, the reference electrode was placed on the contralateral mastoid, so that stimulation effects to bilateral temporo-parietal regions could be observed distinctively.

To allow comparability with the Turkeltaub et al. (2012) study, one of the same measures of single word reading efficiency was employed, the Test of Word Reading Efficiency (TOWRE; Torgesen et al., 1999).

The “spoonerism” task of phonological processing was also chosen as a primary dependent variable as this is a phonological processing task known to be sensitive to skill variation within adults (McCrory et al., 2000). In this task individuals are auditorily presented with two words and asked to swap the first sound of each e.g., “Sunny Terrace” would change to “Tunny Serrace”. To examine pre-post performance changes due to tDCS stimulation it is necessary to have multiple parallel sets of stimuli of equivalent difficulty and so these were custom-made for the study (see Methods for details of stimuli construction). Both accuracy and reaction time can be used as outcome variables for the spoonerism task, however to rule out the possibility that post-stimulation changes in reaction time are due to lower level motor/response factors, a non-phonological decision making task with equivalent response demands was created to act as a control task.

Using these tasks, this study set out to determine the impact upon reading and phonological processing of type of tDCS stimulation (anodal vs. cathodal) on homologous brain regions (left vs. right TPJ).

Given the importance of the left TPJ in reading and its subprocesses (Pugh et al., 2001; Church et al., 2008), we predicted that we might see similar facilitatory effects of anodal stimulation to the Left TPJ, however given the field’s incomplete understanding of the role of the right hemisphere in speech and literacy processing, this part of the study was exploratory in nature.

## Materials and methods

### Participants

This study included 39 healthy right-handed participants (female = 31), ranging in age from 21 to 34 years of age (mean = 26.85; standard deviation = 3.26). All participants were native English speakers, and had an absence of history of reading disability or dyslexia. No participants were taking any central nervous system-active drugs or medications, or had a history or presence of any neurological or psychiatric disorder or disease. For safety reasons regarding the tDCS procedure, potential participants were also screened for pregnancy, seizures, epilepsy, severe headaches, and metallic, electrically sensitive or mechanical implanted devices. The study was approved by the Harvard University Faculty of Arts and Sciences Institutional Review Board and written consent was obtained from all participants.

### Tasks

#### Spoonerisms

This task was designed to assess a participant’s phonological processing skills by using spoonerisms—the act of switching the first sound of two words (e.g., Marble, Balloon : Barble, Malloon). Spoonerisms are usually presented as a production task, i.e., the participant verbally produces the resulting switch and accuracy is scored without regards to speed of response. However, given that performance changes after a 20 min tDCS session are likely to manifest in reaction time, as opposed to accuracy, it became problematic knowing which reaction time variable to use in a production task—the point at which the participant starts their answer or the point at which the second utterance is finished. Given the large inter-individual variability seen in piloting and the technical intensity of capturing these exact points within a spectrogram for each trial item, it was decided to adapt the task to an auditory decision task. In this version, each trial of the spoonerism task included the aural presentation of the first pair of words (e.g., Marble, Balloon), a one-second pause, and then the presentation of a second pair of words that may or may not be the correct spoonerism of that first pair. Once the participant determined whether the second pair of words was, indeed, the correct spoonerism, they were to respond as quickly as possible—pressing the right arrow key if the second pair of words was the correct spoonerism, and the left arrow key if it was an errored spoonerism. Once a response was made, or after a 10 s wait, the program progressed to the next trial. There was a three-second delay at the start of each trial, and trials were presented in random order.

Before the start of the task, the instructions were given both verbally by the investigator and in writing on the computer screen. The task was programmed using PsychoPy, a Python-based interface specifically designed for psychology and behavioral tasks (Peirce, 2009). The stimuli were presented aurally through two speakers attached to the computer, set at either side of the computer screen. During the task, the computer screen had a static image reminding participants which buttons corresponded to the choices. Participants used a key response (right and left arrow keys).

The words used in this task were chosen by creating eight equivalent lists of 15 pairs of bisyllabic words. This process included balancing the word pairs within each list according to their phonemic properties (syllable length, number of consonant clusters, length of vowels and the manner, voicing and place of the initial consonant), as well as their lexical frequency and imageability. All stimuli were recorded by a male with a standard north-eastern US accent using Audacity audio software, and saved as WAV files. The pairs of words were recorded with a stimulus onset asynchrony (SOA) of one second.

In addition to the correct spoonerisms, five different types of errors were created: (1) Removal—The first sound removed from each word (Marble, Balloon : arble, alloon); (2) Second-sound Perseveration—the first sound of the second word is used for both words (Marble, Balloon : Barble, Balloon); (3) First-sound Switch—the first sound of the first word is switched out for a phonetically similar sound (changing just one parameter of place, manner or voice and keeping the other two parameters constant), and that is used for both words (Marble, Balloon : Narble, Nalloon); (4) Second-sound Switch—the first sound of the second word is switched out for a phonetically similar sound, and that is used for both words (Marble, Balloon : Parble, Palloon); and (5) Ending—the ending of each word is switched (Marble, Balloon : Marloon, Balble).

Each stimulus list contained an equivalent number of the different error types. The eight equivalent lists were grouped into 4 sets of two, and within each set, one list was turned into 15 correct spoonerism trials while the other became three trials each of the five error types. This made four equivalent sets of 30 trials, which were used as the four versions of the spoonerism task. The four versions were piloted to ensure equal difficulty and other factors impacting list parity, as well as overall appropriate design. Reaction time per trial was recorded. Any individual trial that was more than 3 standard deviations above or below that participant’s average response time (for that session) was removed from further analysis. A mean reaction time for each set was then calculated per individual.

#### Motor response

This task assessed a participant’s motor response time in order to use it as a control when evaluating the effect of the tDCS stimulation. As the words “Left” or “Right” were heard, participants were to press the corresponding arrow key as quickly as possible. Once a response was made, or after a five-second wait, the program progressed to the next trial. There was a one-second delay at the start of each trial and trials were presented in random order.

At the start of the task, instructions were given both verbally by the investigator and in writing on the computer screen. Also using PsychoPy, the stimuli were presented aurally through two speakers attached to the computer, set at either side of the computer screen. During the task, the computer screen had a static image reminding participants which buttons corresponded to the choices. Participants used a key response (right and left arrow keys). As with the spoonerisms task, there were 30 trials in a set and four alternate sets created. Reaction time per trial was recorded. Any individual trial that was more than 3 standard deviations above or below that participant’s average response time (for that session) was removed from further analysis. A mean reaction time for each set was then calculated per individual.

#### Reading efficiency

The Test of Word Reading Efficiency (TOWRE 2; Torgesen et al., 2011) is a measurement of the ability to read words accurately and fluently. This task was included to see if the tDCS stimulation had any direct effects on reading efficiency. It includes 2 subtests: (1) Sight Word Efficiency—participants have 45 s to read aloud as many of the 108 words as they can; and (2) Phonemic Decoding Efficiency—participants have 45 s to read aloud as many of the 66 pronounceable non-words (e.g., “bremick”) as they can.

There were four alternative versions of this test, each including two subtests (words and non-words) as well as a brief practice before each subtest. During the 45 s that participants were reading the words or non-words, the investigator marked their own copy of the list, making note of any missed or incorrect words. If the participant did not finish the entire list within the 45 s, the investigator marked the last correctly-completed word before the timer sounded. If the participant did finish the entire list within the 45 s, the investigator marked the time of completion. Scoring included total number of correct words as well as calculating the number of correct words per second.

### Transcranial direct current stimulation

tDCS was delivered through a pair of carbon rubber electrodes placed into two saline-soaked sponges (35 cm^2^) and connected to the direct current device (Activa Dose®).The intensity of the current was set to 2 mA for a duration of 20 min with an additional 30 s of ramp up and down periods both in the beginning and at the end of stimulation.

Areas of stimulation: Wernicke’s area (Left/CP5) or its contralateral presentation (Right/CP6), were localized and marked using the 10–20 EEG system reference points. Based on a randomization list either the cathodal or anodal electrode was placed over Left/ CP5 or Right/ CP6 and the remaining electrode was put on the contralateral mastoid and held by elastic rubber bands.

After ten minutes of stimulation, participants were told to start the spoonerism task as described above. For blinding purposes, tests and stimulation were administered by different researchers. Side effects were assessed at the end of each visit.

### Procedure

This was a double-blind, cross-over study where all subjects were randomly assigned to receive tDCS on either the left temporo-parietal junction (CP5/Left, *n* = 19) or its contralateral homologue on the right hemisphere (CP6/Right, *n* = 20).

Each participant took part in two testing sessions—one with an anodal stimulation and the other a cathodal stimulation (the order for each participant was counter-balanced). While it is common within tDCS studies to include a sham condition as a neutral comparison, it was decided here to use each individual as their own control in seeing if there was a significant within-individual difference between anodal and cathodal conditions. The two sessions were spaced apart so as to avoid carry-over effects from the stimulation. This spacing ranged from 5 to 49 days (mean = 14.45; standard deviation = 10.07). The wide range was, in large part, a result of coordinating with participant schedules, but since participants re-familiarized themselves with the tasks during practice sessions at the beginning of the second session, the discrepancy in inter-session time would not be expected to have an impact on performance. Regression analysis for inter-session time showed no significant effect on comparative performance between anodal and cathodal stimulation or for left vs. right groups (*p* > 0.05).

After completing the informed consenting procedure, participants were given an explanation of the spoonerism task. They then completed a practice version of the spoonerism task that consisted of comprehensive instructions and 10 trials using words that were not a part of the experimental versions of the task. The participant began being fitted with the tDCS equipment and this was completed while they worked through the practice items. Once the equipment was fully in place and the participant was given time to ask any remaining questions and clarifications, they began the battery of tasks. All participants followed the same order during each of their two sessions.

Before beginning the tDCS stimulation, participants completed the spoonerism task, motor task and TOWRE. Once the TOWRE was completed, the tDCS stimulation was started, with special attention being given to the participant’s physical experience of the stimulation. During the first 10 min of the stimulation, participants were instructed to sit and relax but to try not to fall asleep. After 10 min of stimulation, the same battery of tasks (spoonerism, motor and TOWRE) was administered in the same order. These tasks were completed shortly before the conclusion of the 20-min tDCS stimulation, typically with approximately 1 min remaining. After the tDCS stimulation was finished and the equipment was removed, a safety screening was conducted to account for possible side effects of the stimulation, before participants left.

Throughout the two sessions, each participant completed four versions of the spoonerism task, four versions of the TOWRE, and the same motor task four times. The sequence in which each participant was assigned the versions of the spoonerism task and TOWRE was balanced between individual participants within each group (left-hemisphere or right-hemisphere). This resulted in the two groups having identical distribution in regards to the sequence of task versions.

### Statistical analysis

Statistical analyses were performed using STATA 12 software (StataCorp. 2011, College Station, TX: StataCorp LP). Given the nested, cross over design and the exploratory nature of the study we divided our analyses into three parts. (i) Since we expected more robust changes with anodal stimulation (Jacobson et al., 2012) we initially tested the effect of HEMISPHERE (left vs. right) only in subjects who received anodal stimulation. (ii) We then tested the effect of POLARITY (anodal vs. cathodal) within each hemisphere. In these two steps, we ran ANOVA models where the dependent variable was the difference between post- and pre- stimulation value; the fixed independent variable was HEMISPHERE or POLARITY and the random variable was ID (for polarity only). The decision to use difference scores as opposed to absolute pre- and post- scores was motivated by the desire to maintain statistical power within the ANOVA models. (iii) Finally to further explore the pre- vs. post- effects of stimulation we also run individual *t*-tests for anodal stimulation within each hemisphere.

## Results

Out of 39 who completed the study 19 subjects received left and 20 subjects received right (CP5/6) stimulation. One subject who was assigned to the left/CP5 group dropped out after completing the first session and was excluded from the analysis. Also one subject in the right/CP6 group was also excluded due to an excess of extreme reaction time outliers in his data. The most common reported side effects were tingling (56%), itching (14%) and mild headache (4%). There were no significant differences between groups (Right vs. Left) for their performance on the first administration of the spoonerisms, motor and reading tasks (*p* < 0.05).

Table 1 shows group means and standard deviations (SD) for all dependent variables, while Table 2 shows the same variables as difference values, as used within the ANOVA models (difference value between post and pre- stimulation values, with standard deviations).

**Table 1:** **Means and standard deviations (SD) for spoonerisms, motor response and reading efficiency**.

		RIGHT (Mean ± SD)				LEFT (Mean ± SD)			
		Anodal		Cathodal		Anodal		Cathodal	
		Pre	Post	Pre	Post	Pre	Post	Pre	Post
Spoonerisms	TCorr	28.37 ± 2.01	28.89 ± 1.49	28.2 ± 1.87	28.4 ± 1.50	27.94 ± 1.7	28 ± 2.35	28.11 ± 1.64	27.78 ± 2.49
	RT	1.88 ± 0.59	1.69 ± 0.39	1.86 ± 0.61	1.74 ± 0.55	2.03 ± 0.52	2.02 ± 0.72	1.95 ± 0.48	1.89 ± 1.54
	CRT	1.82 ± 0.51	1.68 ± 0.39	1.82 ± 0.56	1.72 ± 0.55	1.97 ± 0.50	1.98 ± 0.69	1.93 ± 0.48	1.86 ± 0.50
Motor	TCorr	29.7 ± 0.58	29.5 ± 0.77	29.74 ± 0.56	29.7 ± 0.48	29.5 ± 0.78	29.44 ± 0.92	29.39 ± 0.98	29.39 ± 0.78
	RT	0.56 ± 0.08	0.57 ± 0.07	0.59 ± 0.07	0.59 ± 0.09	0.59 ± 0.07	0.57 ± 0.06	0.58 ± 0.08	0.57 ± 0.06
Reading Efficiency Sight words	TCorr	103.1 ± 7.89	104.26 ± 5.98	104.5 ± 5.7	104.6 ± 6.12	101.12 ± 8.49	99.29 ± 8.39	98.33 ± 9.25	98.89 ± 8.83
	CWS	2.39 ± 0.29	2.46 ± 0.28	2.43 ± 0.23	2.46 ± 0.27	2.40 ± 0.37	2.32 ± 0.39	2.29 ± 0.37	2.28 ± 0.034
Reading Efficiency Nonwords	TCorr	63.32 ± 4.74	63.53 ± 4.43	63.16 ± 3.80	63.68 ± 4.04	61.05 ± 5.59	61.5 ± 5.49	60.78 ± 6.41	60.5 ± 6.28
	CWS	1.48 ± 0.17	1.51 ± 0.17	1.53 ± 0.23	1.51 ± 0.19	1.47 ± 0.30	1.49 ± 0.32	1.43 ± 0.26	1.46 ± 0.31

*Note: TCorr = total correct responses; RT = mean reaction time for all responses; CRT = mean reaction time for correct responses only; CWS = number of correct words per second*.

**Table 2:** **Means and standard deviations (SD) for spoonerisms, motor response and reading efficiency difference scores**.

		RIGHT (Mean ± SD )		LEFT (Mean ± SD )	
		Anodal	Cathodal	Anodal	Cathodal
Spoonerisms	TCorr	0.52 ± 1.46	0.21 ± 1.31	0.06 ± 2.04	−0.033 ± 2.49
	RT	−0.19 ± 0.30	−0.11 ± 0.16	−0.005 ± 0.36	−0.06 ± 0.28
	CRT	−0.15 ± 0.25	−0.10 ± 0.14	0.015 ± 0.34	−0.068 ± 0.25
Motor Response	TCorr	−0.16 ± 1.07	−0.05 ± 0.52	−0.05 ± 0.94	0 ± 1.03
	RT	0.001 ± 0.05	0.001 ± 0.063	−0.01 ± 0.05	−0.008 ± 0.06
Reading Efficiency Sight words	TCorr	1.16 ± 5.29	0.15 ± 3.68	−1.82 ± 4.5	0.55 ± 3.68
	CWS	0.06 ± 0.17	0.03 ± 0.2	−0.079 ± 0.22	−0.008 ± 0.18
Reading Efficiency Nonwords	TCorr	0.21 ± 1.72	0.53 ± 1.87	0.44 ± 1.20	−0.28 ± 1.99
	CWS	0.029 ± 0.11	−0.02 ± 0.10	0.021 ± 0.06	0.03 ± 0.12

*Note: TCorr = total correct responses; RT = mean reaction time for all responses; CRT = mean reaction time for correct responses only; CWS = number of correct words per second*.

To anticipate the key results, the most salient finding was an increase in reading speed following right hemisphere anodal stimulation, relative to other stimulation conditions. In addition, anodal stimulation consistently had a more significant impact compared to cathodal stimulation.

In the first part of the results section we present findings from the ANOVA models for the effects of anodal stimulation over each hemisphere and as compared to cathodal stimulation within each hemisphere. In these models the dependent variable is the difference between post- and pre- values for each given test. In the second part of the results, pre- and post- values are compared with paired *t*-test individually to explore trends further.

### Reading efficiency

The most significant finding yielded by the ANOVA models concerned the differential hemispheric impact of anodal stimulation upon reading efficiency.

The two dependent variables derived from the TOWRE for both the Sight Word and Phonemic Decoding subtests respectively (both analyzed here as difference scores) were (a) correct words read per second (“CWS” in Tables 1, 2); and (b) the total number of words correctly read aloud in 45 s (“TCorr” in Tables 1, 2).

**Figure 1:**
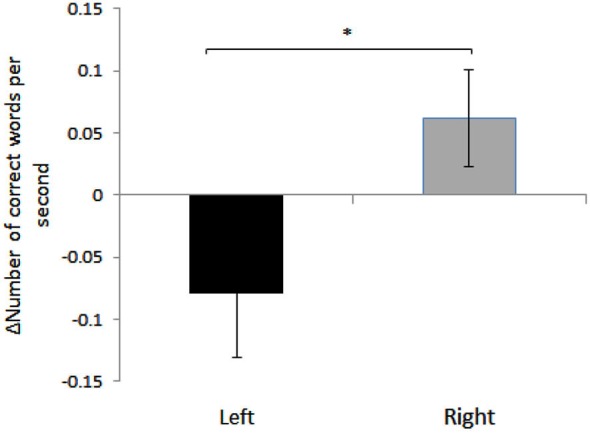
**Mean differences between pre- and post- anodal stimulation for each hemisphere: TOWRE Sight Word Efficiency, number of correct responses per second**. Error bars represent standard error of mean (Mean ± SEM, Left: −0.079 ± 0.05; Right: 0.06 ± 0.03). *Significantly different (*p* = 0.037).

We found a significant effect of HEMISPHERE, with change in the number of correct words per second for sight words being significantly different in participants who received right hemispheric anodal stimulation compared to subjects who received left side anodal stimulation. (Right: 0.06 ± 0.17, Left: −0.079 ± 0.21, Mean ± SD; *F*_(1,35)_ = 4.71, *p* = 0.037). See Figure 1. A negative difference score for correct words per second equates to a lower number of correct words per second post-stimulation. Therefore, while reading speed slightly increased for the participants who underwent right hemisphere anodal stimulation, the opposite was true for the left hemisphere group.

There was also a trend towards significance in difference scores for the total number of sight words read correctly in 45 s (Right: 1.15 ± 5.29, Left: −1.82 ± 4.5, Mean ± SD; *F*_(1,35)_ = 3.27, *p* = 0.079 ). Again, the direction of this trend indicates that after anodal stimulation, there were more words read by the right hemisphere group, whereas the left hemisphere group read less compared to their pre-stimulation baseline. Across hemispheres, there was no significant effect of POLARITY.

No significant effect of HEMISPHERE or POLARITY were found for phonemic decoding (non-word) speed or accuracy.

### Spoonerisms

For Spoonerisms performance, three dependent variables were derived—the total number of correct responses (“TCorr” in Tables 1, 2), mean reaction time for correct responses only (“CRT” in Tables 1, 2) and mean reaction time across both correct and incorrect trials (“RT” in Tables 1, 2). There were no significant effects of HEMISPHERE or POLARITY for any of the dependent variables.

### Motor response

No significant effects of HEMISPHERE or POLARITY were found.

### Exploratory analysis. (Pre vs. Post Paired *T*-Test)

In order to follow-up the significant results above, a series of paired sample *t*-tests were carried out. The above results informed us that comparing the right hemisphere group to the left hemisphere group, anodal stimulation appeared to have differential effects, with more performance-enhancing effects seen in sight word reading for the right hemisphere group. We wanted to complement this between-subject analysis with a within-individual analysis, to see if a similar pattern was found. To constrain the number of comparisons, we restricted this analysis to the anodal stimulation condition, where we had seen the greatest effects.

In the right hemisphere group there was a significant difference between pre- and post- values for Spoonerism task total reaction time (RT: *p* = 0.0138) and reaction time for correct responses (CRT; *p* = 0.0173; see Table 1 for mean values) after anodal stimulation.

No significant effect was observed for the other measures, or for the left hemisphere group.

### Analysis of spoonerism detection by error type

As a final step, the detection of spoonerisms by error type was examined, to see if mistakes made on a certain error type were more common than others (see Table 3). The aim of this analysis was (a) to verify the validity of the measure; and (b) to see if specific aspects of phonological processing that might be impacted by tDCS stimulation. The largest number of detection errors were made for the “Ending” stimuli (Marble, Balloon: Marloon, Balble), though there was no significant difference between error type. All participants were also debriefed after carrying out the spoonerism task, to see if a consistent strategy was used. Overall, participants reported a wide range of strategies for performing the task. The most common strategy was mentally carrying out the spoonerism in the pause between the stimulus words and the following word pair, and then seeing if the second word pair matched the mental answer (40% of participants). A related strategy and the second most common (24% of participants) was listening to the stimulus words, holding the reversed initial phonemes in mind and then seeing if these matched those in the second word pair presented.

**Table 3:** **Means and standard deviations (SD) for detection accuracy for different spoonerism error types. See method section for further description of error types**.

	RIGHT (Mean ± SD )				LEFT (Mean ± SD )			
	Anodal		Cathodal		Anodal		Cathodal	
Error type	Pre	Post	Pre	Post	Pre	Post	Pre	Post
**Removal**	3 ± 0	3 ± 0	2.95 ± 0.23	2.95 ± 0.23	2.94 ± 0.23	2.78 ± 0.55	2.89 ± 0.32	2.89 ± 0.32
**Second-sound Perseveration**	3 ± 0	3 ± 0	2.9 ± 0.23	2.9 ± 0.3	2.89 ± 0.32	3 ± 0	3 ± 0.30	2.94 ± 0.23
**First-sound Switch**	2.9 ± 0.23	2.95 ± 0.23	2.95 ± 0.23	2.95 ± 0.23	3 ± 0.3	3 ± 0	2.89 ± 0.32	2.89 ± 0.32
**Second-sound switch**	2.95 ± 0.22	2.95 ± 0.23	2.95 ± 0.23	2.95 ± 0.23	2.89 ± 0.32	2.89 ± 0.32	2.89 ± 0.32	2.84 ± 0.51
**Ending**	2.7 ± 0.80	2.9 ± 0.31	2.9 ± 0.31	2.95 ± 0.23	2.55 ± 1.04	2.72 ± 0.75	2.78 ± 0.54	2.77 ± 0.54

## Discussion

This study set out to explore the effect of tDCS stimulation on reading and phonological processing. There were two key findings. Firstly, the largest hemispheric differences in response were seen for sight word efficiency, following anodal stimulation. In this case there was a significant difference between the right and left hemisphere stimulation groups, with performance decrement seen for the left hemisphere group, and mild performance increase seen for the right hemisphere group. Secondly, when these findings were followed up to look at the significance of pre-post stimulation change within individuals, again, anodal stimulation to the right hemisphere yielded the strongest results, with spoonerism reaction time increasing significantly from pre- to post- stimulation. Reaction time did not change in the parallel motor control task for this condition (or any other), suggesting that the improvement in spoonerism task performance was not due to more general motor/response processes.

At first glance, these findings are surprising given the known dominance of the left hemisphere in language processing, and indeed, were contrary to our initial predictions. It is feasible to speculate that we would see most behavioral change when stimulating a cortical area known to be specialized in the observed behavior. On the other hand, neuromodulatory techniques such as tDCS and Transcranial Magnetic Stimulation (TMS) are increasingly demonstrating the complex interplay between hemispheres. For example, one phenomena that has been increasingly reported with tDCS as compared to TMS is that of defocusing (Fregni et al., 2008; Boggio et al., 2009). Given that the effect of tDCS is relatively more diffuse than that of TMS, an explanation that has been speculated to explain differences in effect is that the more widespread excitatory effect of anodal tDCS could result in unnatural competition from surrounding areas, thus reducing efficiency at the main processing site for a given skill i.e., defocusing. This would potentially explain why some of the hemispheric differences in stimulation response in this study appeared to be driven more by reduction in performance of the left hemisphere, as compared to improved performance of the right hemisphere.

It is possible to speculate that if an individual’s left hemisphere phonological system is already functioning at an optimal level, there is less scope for stimulation to result in further improvement. Indeed, there is strong evidence that tDCS effects differ between healthy participants and those with an impairment (Suzuki et al., 2012). In contrast, the activity of the right hemisphere may have more potential for enhancement. There are precedents for such an explanation in other processing domains. For example, investigating motor functioning within a group of healthy adult volunteers, Boggio et al. (2006) applied anodal and sham stimulation to dominant (left) and non-dominant (right) hemisphere primary motor cortex respectively. Enhancement of motor function was only seen for anodal stimulation of the non-dominant hemisphere. The authors suggest a ceiling effect for the already optimized dominant hemisphere, an explanation that may also hold here.

Relating these findings to the most similar study to date, that of Turkeltaub et al. (2012), the results appear discrepant. Comparing post-stimulation differences after sham vs. anodal stimulation, Turkeltaub et al. found one key effect, that of increased real word reading efficiency (accuracy/time) on the TOWRE (*p* < 0.034) after anodal stimulation of the left hemisphere, compared to sham. Note however, that reading was measured only post-stimulation, so the inference is that slightly different reading performance on two separate days was most likely attributable to the stimulation alone, and not any other factors that could cause performance variability between those days. However, this inference is impossible to confirm.

More broadly, we might expect the effects of stimulation to look slightly different between the studies due to differential electrode placement. While the positioning of the active electrodes was closely equivalent (CP5/6 in this study and midway between T7/TP7 in Turkeltaub et al.), the current study used the contralateral mastoid for the reference site, while Turkeltaub et al. used the right hemisphere homologue (T8/TP8). This means that while the path of current flow from the anode to the cathode in each study likely overlapped, the reference electrode in the current study was positioned in a relatively inert position, whereas in the Turkeltaub et al. study, the reference electrode was positioned to result in cathodal flow directly at the right hemisphere temporal parietal junction (TPJ). Given the unresolved questions about the role of the right TPJ in phonological processing discussed above, and the positive change in phonological processing observed in this study as a result of anodal right TPJ stimulation, it is important to understand that the Turkeltaub et al. results were a result of simultaneous anodal left and cathodal right hemisphere stimulation, and so cannot be attributed directly to the anodal left hemisphere stimulation alone.

Many questions remain and cautions are needed in relation to the speculated mechanisms at work in the current study. To argue that performance change is seen after anodal right stimulation because the right hemisphere has more scope for performance change also begs the question of why cathodal stimulation to the right hemisphere did not result in a decline in performance, in the way that cathodal stimulation has often been reported to act (Purpura and McMurtry, 1965; Nitsche and Paulus, 2000). One explanation may lie in the recent review of Jacobson et al. (2012). These authors demonstrate that the assumption of annodal stimulation causing enhancement of cortical excitability during stimulation while cathodal stimulation results in the opposite, is commonly substantiated in motor functions, but it is much rarer in cognitive studies. Further replication of this work is clearly needed.

To conclude, this study demonstrates preliminary evidence concerning the stimulability of reading efficiency and phonological processing with tDCS. Although the behavioral changes observed here were modest, while a significant proportion of individuals fail to respond to existing best practice for reading instruction (Torgesen, 2000), it behooves us to seek new solutions to the inherent difficulty of learning to read. This finding generates questions concerning the application of tDCS to individuals with documented difficulties in phonological processing e.g., those with developmental dyslexia. tDCS is still being applied cautiously within pediatric populations, however progress in this area is rapid and positive (Kessler et al., 2013; Andrade et al., 2014).

## Conflict of interest statement

The authors declare that the research was conducted in the absence of any commercial or financial relationships that could be construed as a potential conflict of interest.
